# Reply to: Questioning whether the IgM Fc receptor (FcμR) is expressed by innate immune cells

**DOI:** 10.1038/s41467-022-31226-2

**Published:** 2022-07-11

**Authors:** Shawn P. Kubli, Parameswaran Ramachandran, Gordon Duncan, Rich Brokx, Tak W. Mak

**Affiliations:** 1 Princess Margaret Cancer Centre, 610 University Avenue, Toronto, ON M5G 2M9 Canada; 2 Department of Medical Biophysics, University of Toronto, 101 College Street, Toronto, ON M5G 1L7 Canada; 3 Department of Immunology, University of Toronto, 101 College Street, Toronto, ON M5G 1L7 Canada; 4 Department of Medicine, University of Hong Kong, Pok Fu Lam, Hong Kong

**Keywords:** Cancer, Tumour immunology

**replying to** Skopnik et al. Nature Communications 10.1038/s41467-022-29407-0 (2022)

The Matters Arising submission from Skopnik et al. is a comparative analysis of single-cell RNA sequencing (scRNAseq) data obtained from two groups^1,2^. The submission highlights the well-documented limitation of scRNAseq technology, termed “dropout events”, that result in zero detected reads for some genes despite alternative evidence of their expression^3–5^. The submission further debates a hypothesis relating to Fcmr expression within the hematopoietic system. The authors highlight negative data for Fcmr expression in myeloid cells as it relates to reported expression data in innate immune cells from various groups. This debated point of Fcmr expression is further detailed in a review by Wang et al. ^6^.

To investigate the nature of Fcmr expression within the hematopoetic system, Skopnik et al. ^2^ focused their investigations to the scRNAseq we performed on tumor infiltrating mononuclear phagocytes and compared these data with their own data from splenic B cells. We value the opportunity to revisit these data and appreciate their comments. While intrigued by the re-analysis of our data and the hypotheses generated, the model in our publication is based on a combination of functional and genetic experiments presented therein^1^, and not concluded based on a single data source—scRNAseq—alone. The scRNAseq data in our paper was used to shape our understanding of the impacts Fcmr gene loss has on innate immune cells in the TME. Here we briefly discuss the following: (1) technical limitation in interpreting surface receptor expression in our scRNAseq data, (2) the biological and technical limitations for comparing the two different scRNAseq data sets, and (3) the genetic and functional experiments from our manuscript that need to be considered for generating hypotheses relating to mechanisms of Fcmr expression and function in innate immune cells.

As with any technology, scRNAseq has its limitations. Skopnik and colleagues’ re-analysis of our data highlights a well-characterized limitation of scRNAseq data that relates to the shallow sequencing depth associated with the 10X platform^3–5^. While the 10X scRNAseq platform has the benefit of increased number of individual cells sequenced, this comes at the cost of sequencing depth that may result in a gene drop-out effect^3,4^. Skopnik et al. focus on read counts for Fcmr, as well as four other surface receptor genes which are either common or unique to B and innate immune cells. We complement their analysis by comparing read counts from our scRNAseq data to protein expression from flow cytometry data for those cells sorted for sequencing (Fig. 1). While Skopnik et al. chose random surface receptor genes, in Fig. 1, we show read counts and expression-overlaid tSNE plots for those surface receptors we used to sort cells for sequencing. All 14,409 cells sorted had positive to high level protein expression of *Mertk*, *Fcgr1*(CD64), *Itgam*(CD11b), and *Adgre*(F4/80). However, as indicated in the tSNE plots and histograms, there is a large discrepancy in the transcript read counts for these markers. For example, sorted cells were all MerTk^+^ but the average UMI count was only 1 (7159 cells had a UMI count of 0 for *Mertk*) (Fig. 1a, b). This means MerTk transcript was undetectable for 49.7% of the cells (14,409 total cells) even though all cells show protein level expression. In addition, we examined two additional surface receptor genes, *Itgax* (CD11c) and *Ly6c1* (Ly-6c) which are both expressed in a large portion of sorted cells at the protein level but lack significant detection in our scRNAseq data (Fig. 1c). While the lack of read counts for Fcmr may represent a lack of expression, the dropout limitations of the 10X platform must be considered when formulating hypotheses relating to this data. While a reductive interpretation of the scRNAseq data might suggest the sorted innate immune cells do not express Fcmr, our published work considered the transcript data as part of a broader context of genetic and functional experiments that facilitated our interpretation that Fcmr has a direct cell-intrinsic role in innate immune cells within the TME. Additionally, there are a variety of well documented factors in complex biological systems, including RNA processing/stability and transcription/translation kinetics, which influence a non-linear relationship between the transcriptome and proteome (reviewed in refs. ^7,8^).

**Fig. 1:**
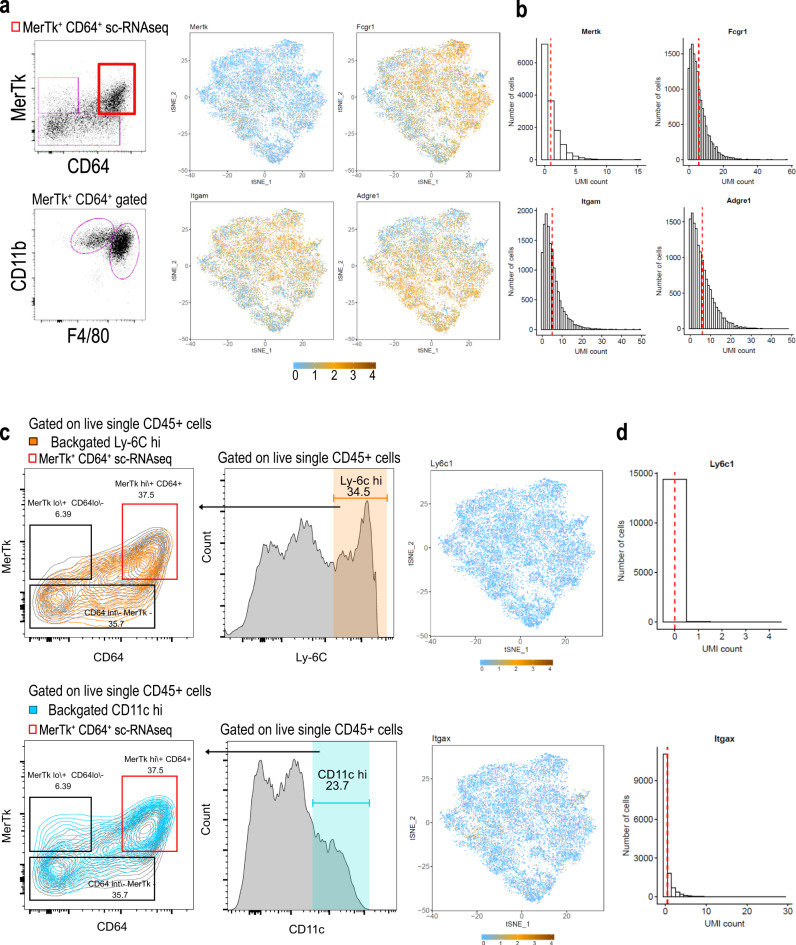
Discordance in flow cytometric protein data and scRNAseq read counts. **a** Flow cytometry dot plots (left) illustrate the population of MerTk^+^ CD64^+^ (bold red gate) tumor mononuclear phagocytes (TMP) that were FACS-sorted for scRNAseq (top left), and this populations CD11b and F4/80 expression (bottom left). The corresponding transcript data for the markers shown in the flow plots, which was obtained from scRNAseq analysis, is overlaid on t-SNE plots for each of the four genes that encode those markers; Mertk (MerTk), Fcgr1 (CD64), Itgam (CD11b), and Adgre (F4/80) (Right). These t-SNE represent cell clusters from FACS-sorted TMP isolated from B16 melanoma tumors growing in *Fcmr*^+/+^ and *Fcmr*^−/−^ mice. Gene expression is overlaid on these tSNEs to highlight differential read counts for each of the four genes, where a UMI of 0 is illustrated in blue. **b** Histograms illustrating the distributions of raw UMI counts per cell for those same four genes (*Mertk*, *Fcgr1*, *Itgam*, and *Adgre*) which were stained at the protein level during FACS-sorting of TMP. Mean count values are denoted by a vertical red dashed line. **c** and **d** (Left) The bicolor contour plots illustrate surface receptors Ly-6c and CD11c backgated from histogram plots (middle), where the Ly-6c^hi^ is gated in orange (top) and the CD11c^hi^ is gated in blue (bottom). Apparent from the backgating (left), both surface receptors Ly-6c (*Ly6c1*) and CD11c (*Itgax*) are expressed at the protein level on the TMP that were sorted for scRNAseq. (Right) tSNE expression-overlaid plots for *Ly6c1* (top right) and *Itgax* (bottom right) show zero and near zero cells with transcript for these receptors. UMI of 0 is illustrated in blue. **d** Histograms illustrating the distribution of UMI for *Ly6c1* and *Itgax*, as in **b** above.

Innate immune cell expression of Fcmr is already well documented^9–14^, and was therefore not the focus of our experiments. However, we do have some preliminary flow cytometry data from peripheral blood monocyte staining from a discontinued rat anti-mouse-Fcmr antibody project (Fig. 2). While Honjo and colleagues argue a lack of Fcmr protein on innate immune cells as evidenced by differences in detection with different mAb clones^10,15^, recent evidence suggests that cell-specific post-translational modification can alter mAb detection of surface receptors in a cell-dependent manner^16^. Furthermore, Fcmr harbours well documented post translational glycosylation^17^. Taken together, the conflicting results from previously published work^9–15^ could relate to antibody paratope differences and/or immune cell-specific differences in Fcmr glycosylation.

**Fig. 2:**
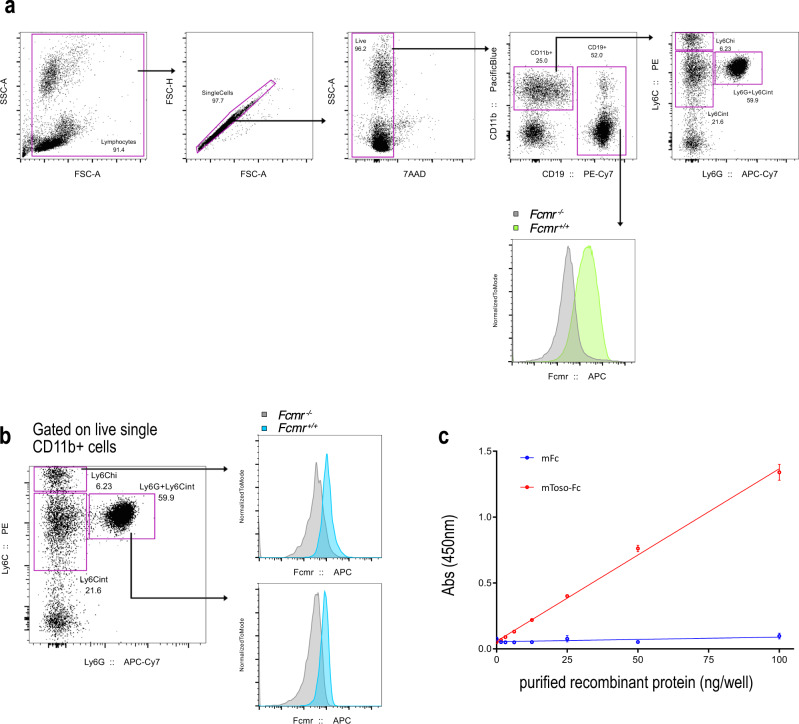
Fcmr is expressed in classical monocyte and neutrophil innate-immune cells. **a** (Top) Gating schematic for peripheral blood staining implementing an in-house anti-mouse-Fcmr antibody. (Bottom right) Histogram overlay of Fcmr staining of peripheral blood B cells in *Fcmr*^+/+^ (green) mice where *Fcmr*^−/−^(gray) serve as a negative control. **b** (Left) Dot plot illustrating peripheral blood CD11b + monocyte and neutrophil populations. (Right) Histogram overlay of Fcmr staining of peripheral blood innate immune cells in *Fcmr*^+/+^ (blue) mice where *Fcmr*^−/−^ (gray) serve as a negative control. **c** Direct ELISA illustrating specific recognition of the extra-cellular domain of mouse Fcmr with in-house α-mouse-Fcmr antibody. Technical triplicates are represented with SE.

Aside from the technical limitations associated with scRNAseq data, there are additional technical and biological factors that may limit the degree to which scRNAseq data can be used for direct comparative expression analysis between independent studies. Important factors to consider are the sequencing platform and the methods employed for cell extraction and preparation prior to sequencing. A variety of technical factors at various stages of tissue preparation and sequencing can influence the degree of comparison that can be drawn. Making the generous assumption that these technical factors do not impinge on our ability to draw comparison between the two data sets, it is still crucial to consider that these are different cell types, isolated from different tissues, under different pathogenic conditions. In addition to these caveats, previous reports illustrate that B cells may harbor 10–50 times more Fcmr transcript compared to innate immune cell types^13^.

Further to the aforementioned technical and biological considerations, there are a series of genetic experiments accompanying our scRNAseq data that ultimately led us to the main conclusions of our study^1^. More specifically, the following three in vivo genetic experiments illustrate the importance of Fcmr in myeloid cells, and not B cells, during tumorigenesis: (1) myeloid lineage *Fcmr* knockout mice (Fcmr^fl/fl^ LysMCre^+^) exhibit reduced tumor growth compared to Fcmr sufficient littermates (Kubli et al. Nat Commun, 2019, Fig. 1g), (2) B cell lineage *Fcmr* knockout mice (Fcmr^fl/fl^ Mb1Cre^+^) show no differences in tumor growth compared to Fcmr sufficient littermates (Kubli et al. Nat Commun, 2019^1^, Supplementary Fig. 1d), and (3) the adoptive transfer of *Fcmr*^−/−^ moDCs results in reduced tumor growth in wildtype hosts, but not adoptive transfer of littermate *Fcmr* sufficient moDCs (Kubli et al. Nat Commun, 2019^1^, Fig. 5g, h). We further supported the findings from these in vivo studies with in vitro functional and biochemical analysis that illustrate a myeloid cell intrinsic role for *Fcmr*.

How Fcmr–ligand interactions signal within innate immune cells to instruct their maturation is outside the scope of our published work, and an area of ongoing investigation for our group. Based on the findings discussed above we hypothesize Fcmr may be expressed at a level below the limits of detection for our scRNAseq analysis, or that Fcmr is transiently expressed during monocyte maturation. We find the hypotheses and speculations generated by Skopnik et al. ^2^ to be intriguing. However, there are other biological considerations and technical limitations which may account for the lack of observed sequence reads for *Fcmr* in the published data. While the transcriptomic comparative analysis performed by Skopnik et al. supports their hypothesis, there are a variety of other data which support an alternative explanation to their interpretation of the findings. Detection of *Fcmr* transcript from these different cell types, that were isolated from very different tissues, which have varying transcript levels in naïve mice, are not directly comparable. We have previously illustrated Fcmr protein expression in the myeloid lineage^9,10^, for which other groups have independently corroborated expression in both rodent and human myeloid lineage cells^11–14^.

## Methods

### Single-cell sequencing analysis

Tumor mononuclear phagocytes (TMPs) were isolated via FACS and processed using standardized 10X Genomics Reagent Kit protocols. The raw sequenced reads were subjected to the CellRanger bioinformatics pipeline v2.0.1. The assembled UMI count matrix was processed using the Seurat R package v2.1.0 for quality filtering, normalization, clustering, and further downstream analyses. The final matrix contained counts for 16112 genes in 6352 Fcmr^+/+^ and 7999 Fcmr^−/−^ cells. The expression-overlaid tSNE plots shown in Fig. 1 were produced using the FeaturePlots module of Seurat. The UMI histograms were produced using the raw UMI counts corresponding to the full set of cells before quality filtering.

### α-mouse Fcmr antibody

Rat anti-mouse Fcmr sera were generated at an external vendor. In brief, four Lewis rats were immunized with purified recombinant mouse Fcmr-Fc protein^1^. After three subsequent immunizations of 0.1 mg each, serum was collected from each animal. To evaluate efficacy of rat immunization in producing specific and robust B cell responses to mouse-Fcmr, sera from these rats was used for ELISA and flow cytometry experiments. These data (Fig. 2) were subsequently used to inform on selection of two rats for splenic harvest and hybridoma generation. Although anti-Fcmr antibody titres were high in rat sera, this project ultimately failed to produce monoclonal antibodies of the desired affinity.

### Flow cytometry

Peripheral blood was collected into heparinized capillary tubes via tail venipuncture and ejected into 5 ml polystyrene FACS tubes containing 100 μl PBS supplemented with 1% bovine serum albumin (BSA) plus 2 mM EDTA (referred to as FB buffer). Blood was collected from both *Fcmr*^−/−^ mice and *Fcmr*^+/+^, where *Fcmr*^−/−^ served as the negative staining control. Peripheral blood in FB was pelleted at 300×*g* for 5 min at 4 °C, resuspended in 100 μl FB, and incubated for 10 min at 4 °C with 75 μl Fc Block (αCD16/32 2.4G2, Tonbo Bioscience; rat serum, StemCell) in FB containing DNase I (Roche). Rat polyclonal α-mouse-Fcmr was then added at a dilution factor of 1:100 and incubated for 30 min at 4 °C. Stained cells were washed via centrifugation in 2.5 ml FB at 300×*g* for 5 min at 4 °C, and resuspended in 100 μl Fc Block. 100 μl anti-rat secondary was then added to a final concentration of 1:200, and incubated for 30 min at 4 °C. Cells were again washed via centrifugation in 2.5 ml FB at 300×*g* for 5 min at 4 °C, resuspended in 100 μl FB, incubated for 10 min with 75 μl concentrated Fc Block. Following 10 min block, concentrated surface staining antibody cocktail (8x; 25 μl)^1^ was added to a total volume of 200 μl and incubation continued for 30 min at 4 °C. Cells were again washed via centrifugation in 2.5 ml FB at 300×*g* for 5 min at 4 °C. Stained and washed whole blood was then subjected to fixation and red blood cell lysis. For this, pelleted whole blood from final wash was vortexed, resuspended in 1× Lyse/Fix Buffer (BD Biosciences, cat no. 558049), and incubated for 10 min. Cells were then pelleted (300×*g* for 10 min at 4 °C), supernatant discarded, and washed 2× via centrifugation at 300×*g* for 10 min at 4 °C with 2 ml FB. Cells were finally resuspended in 300 μl FB and analyzed by flow cytometry (Fortessa, BD Biosciences).

### ELISA

Wells were coated with either mouse Fc (mFc), or mouse Fcmr-Fc (Toso-Fc) purified recombinant protein^1^, blocked, and washed. Rat polyclonal α-mouse-Fcmr was then added, incubated overnight at 4 °C. Plates were washed, incubated overnight at 4 °C with biotinylated α-rat secondary. Plates were again washed, and incubated with SA-HRP, washed, and bound antibody detected via TMB substrate. Plates were read at 450 nm.

## Data Availability

The authors declare that all data supporting the findings of this study are available within this article or from the corresponding author upon request. Single cell RNA-sequencing data supporting the findings which were generated for this matters arising are available in a public repository; Gene Expression Omnibus database, accession code GSE130287.
